# Temperance

**Published:** 1878-10

**Authors:** 


### HALL'S JOURNAL OF HEALTH.

OUR LEGITIMATE SCOPE IS ALMOST BOUNDLESS: FOR WHATEVER BEGETS PLEASURABLE AND HARMLESS FEELINGS, PROMOTES HEALTH; AND WHATEVER INDUCES DISAGREEABLE SENSATIONS, ENGENDERS DISEASE.

VOL. 25] OCTOBER, 1878 [NO. X.

### WESTMINSTER REVIEW vs. TEMPERANCE.

Divesting the subject of all that is extraneous, we wish to offer our own views on this important question, in as concise and pointed a manner as may be, only remarking, that we are not, nor ever have been, theoretically or practically, connected with any teetotal movement or society whatever, nor do we expect ever to be; yet we, in common with others, feel an interest in the prevalence of true views on all subjects; for only in proportion as truth prevails, can any community be prosperous and happy.

The point in the Review is, *Alcohol is not only not poison, but food*—educing therefrom the general practical fact, that *the moderate use of spirituous liquors is nutritious and healthful.* All that the writer says is predicated on the chemical analysis of *pure* alcohol, of *pure* brandy, of *pure* wine, &c. But granting for a moment that all he says is scientifically and literally true, the broad fact, which no well read man will deny, that pure liquors are not used, except by the very fewest of the few, because they are not to be purchased or secured in any way, unless by men of large fortune or high position—nullifies his argument in its actual application to the masses, for whose good the temperance movement was instituted. We give a single fact out of many, merely as an illustration.

"The Inspector, under the Prohibitory Law, at Buffalo, New York, Dr. H. Cox, has inspected 76 qualities of various liquors since he has been in office. He finds some pure liquor, a great deal of low per centage, and the balance pernicious and drugged concoctions. In domestic brandy and port wine he has found the following ingredients, in large quantities, viz.:

*Prussic acid*, *sulphuric acid*, cider, alum, beet-root juice, (coloring,) *nitric acid*, *logwood*, *lead*, and *copper!* He inspected a cask of liquor represented as domestic brandy, which was very strongly tinctured with sulphuric and nitric acid."

What may be predicated on a pure article, cannot be safely predicated on one which is impure. What may be said of pure air cannot be said of impure air; the man who does it practically, dies! The Review says "*Alcohol is not a poison.*" The ancient Greeks used the same word to express poison and medicine. Physiologically speaking, poisons are substances which derange the vital functions, and produce death by an action not mechanical, of which the most familiar illustrations are Prussic Acid, Laudanum, Strychnine, Nitric Acid, &c. &c. That the common drunkard dies from a derangement of the vital functions, caused by drinking spirituous liquors, the *Review* will perhaps not deny. If, therefore, when immoderately used, it produces undeniable poisonous effects on the animal economy, the question for all practical purposes is lost.

The idea, evidently in the mind's eye of the writer is, *The steady, uniform, and moderate use of spirituous liquors is nutritious and healthful;* but in the concluding sentence of his elaborate article, he exhibits his consciousness of the fact that he has all the time been "*beating the air*," that he has been advocating a theory utterly impracticable in its bearings on the multitude, saying "It is a very dangerous, tricksy spirit, needing the power of a Prospero to make it beautifully obedient; needing sagacity and self-command to make it a blessing." How few have this power, this sagacity, this self-command, let the tens of thousands liquor-lost every year, make reply. But the Reviewer is not willing to be judged by the evident actual tendency of his article; even he has not the boldness to act as the champion for the habitual use of liquor. Says he "the point at issue is not *habitual* over excitement, but *occasional* excitement; we are not considering the case of a man who vitiates his organization by taking stimulus twenty times a day, but of a man who takes it once or twice a day." After all, then, the whole force of the argument is expended on the difference, physiologically speaking, between "poisoning" and "vitiating the organization:" he admits that taking liquor twenty times a day "vitiates the organization," what then is the difference in this connection between "vitiating" and "poisoning?" In law, "*to vitiate*" an obligation or writing means its legal destruction. In ethics, to vitiate a man's morals is destructive of that morality, for then he is moral no longer. In physiology, to vitiate the blood, to poison the blood, are in a free meaning, synonyms. To vitiate, physiologically speaking, is to render incapable of natural action; poisoning does the same thing; vitiation here will as certainly cause death, if kept in action, as will poison; therefore, for all practical purposes, the whole force of the article in the Review narrows itself down to a quibble, the difference between tweedle-dum and tweedle-dee. It is a battle of mere words, a mere flourish of trumpets. It is not for the splitting of a hair that so many of the greatest minds in the nation have waged the temperance war; they do not fight for a name or a phrase; their object is to prevent the destructive effects which follow the use of spirituous liquors, and which the Reviewer acknow ledges and calls "vitiating the organization;" which, if words have any meaning at all, is nothing more than rendering incapable of performing the natural functions, and of which, if continued, a man must of necessity die. It kills the man. Poison can do no more. It kills the man, and that is what temperance men mean when they use the word "Poison."

While, in effect, and to all intents and purposes, the Reviewer admits what the friends of Temperance contend for, we are bold to say that "the occasional excitement of a man who takes liquor once or twice a-day," and for which the Reviewer contends as being beneficial, is a physiological untruth. It is not merely the man who obviously takes liquor "twenty times in a day," nor the habitual drunkard, who is destroyed by it, whose organization is vitiated thereby, but their organizations are inevitably and under all circumstances vitiated, who drink spirituous liquors "once or twice a day."

Any man who drinks liquor regularly every day once, or twice, in the sense meant by the Reviewer, although he may never have been known to be actually drunk, or to have taken it oftener "than once or twice a-day," (which by the way we may well set down as an impossible supposition, and thus a most unfair weapon in argument, yet ruthlessly wielded by the Reviewer,) must have his organization vitiated to the extent of such use; for what is said of a whole may be said of a part, so large as ten per cent.; if drinking "twenty times a-day" "vitiates" acknowledgely, drinking "once or twice a-day" must "vitiate" some.

But of those people who were "never known to be drunk," except to themselves, who "take their dram once or twice a-day," as the saying goes, what will educated physicians of eminence and character of any nation say? We will make no sweeping assertion, will employ no extravagant hyperbole— we will only state that it is of common occurrence, in the practice of such, to see men and women dying from Livers engorged or ulcerated, from stomachs whose coats are thickened and otherwise rendered morbid, and from intestines, powerless to draw nutriment from the food eaten, ending in inanition or chronic diarrhœa, as the unquestioned result of a daily stimulant. Any city practitioner of ability and observation knows full well, that of the many who are reported to die of "*disease of the heart*," no small moiety perish thus suddenly, from the fact that the accustomed stimulus had lost its power, whether that stimulus had been brandy, opium, or tobacco. But let it be borne in mind that the majority of those who are thus benevolently chronicled to have "died of disease of the heart" are not of the common topers; we don't, for the most part, think it worth while to inquire or to state what they die of, but they are men of wealth, retired merchants, bankers, ship-merchants, and the like men, who can command the purest and best liquors in the world.

The Review narrows down the whole question to the broad statement that

"ALCOHOL IS NOT POISON, BUT FOOD."

His mode of argument is literally thus:

1. Food cannot be poison.
2. Food is that which gives force, motive power.
3. Alcohol gives motive power.
4. Therefore alcohol is food, and hence cannot be poison.

The error in this ratiocination is, in assuming for a fact what is a physiological untruth, to wit, that what gives force, motive power, is essentially food. Let us examine this statement.

By a parity of reasoning, we may easily arrive at the reductio absurdum that arsenic is food.

ARSENIC IS NOT POISON, BUT FOOD.

1. Food cannot be poison.
2. Food is that which gives force, motive power.
3. Arsenic gives force, motive power.
3. Therefore arsenic is food, and hence cannot be poison.

It is an uncontradicted fact, that the mountaineers of Hungary and Alsatia are able, by eating a small piece of arsenic, to perform feats of mountain scaling with an ease, and vigor, and success which, without the arsenic would be impossible; but having used it for a while, a premature death is inevitable; and the more speedy, if its employment is abandoned.

That alcohol is not a poison, but food, is the purest conjecture, built on the fallacy that food is force. Such is not its definition. As well might we say that the laughing gas was food, for under its influence one man has the strength of numbers. A more proper definition, and, as far as we can now see, unobjectionable, is, food is that whose continued use, alone, sustains life, maintains health, and supplies muscular power. The continued use of only alcohol does neither, but will inevitably and in every case cause death, within ten days.

We have not the space to join in the side issues, which are mere child's play; they are arguments more striking than true; such as that in Frankfort, Germany, the hotel keeper found that the members of the Peace Congress, who were mostly tetotallers, ate so much of solid food as to create an unheard of deficiency in certain dishes, as compared with an equal number of his countrymen, who revelled in wines, brandies, and lager-beer. If this proves anything, it shows that temperance secures a good appetite; but whether these eating men would not be able to endure an amount of physical exertion far greater than the drinking men—which by the way no intelligent man will deny—it did not suit the argument of the writer to state. There are many such ad-captandum statements running through the whole article, which the most superficial examination would be sufficient to explode, but which have their effect on the casual, unwary, unthinking reader—as, alas, most readers are.

But let us grapple more closely. The argument is that one object of eating is to keep warm; that alcohol is better calori fic food than starch or sugar, and seven times better than beef-steak—that is, that of equal proportions of lean flesh and alcohol, the alcohol has seven times more hydro-carbon, the heat-forming principle. Theoretically, it is true, but the falsity ot the bare statement may be shown by taking two men, in all respects equal; give one fifty pounds of beef-steak, and nothing else but air and water, and give the other fifty pounds of alcohol and nothing else but air and water, which of the two will keep off the cold chill of death the longer? Let common sense answer. The fact is, alcohol warms, and warms only, and man cannot live on warmth alone—while beef-steak both warms and nourishes, as does all food; and what does not do both, cannot sustain life, and cannot be properly denominated food.

But it is not safe to say that because alcohol supplies warmth largely, that it should be used for that purpose. It has for aught we know other constituents than hydro-carbon; chemistry may not detect them, still they may be there. Chemistry does not detect in a hogshead of concentrated marsh-miasm one single atom of a death-dealing quality; cannot absolutely detect anything beyond the constituents of the ordinary air around us; yet the man who breathes it dies. And as the man who breathes this malaria alone, will perish, although chemistry can detect nothing deadly there; and yet when we see the man die who breathes it, we know that it must be deadly! so if we see a man relying on drinking alcohol to keep him warm, and yet see him grow cold in death, we cannot but conclude there is something deathly in alcohol, although chemists cannot detect it.

The fact is, chemistry, notwithstanding all its triumphs, is yet too much in its infancy to afford physiological demonstrations; hence, for the present, we must make use of the truths she has vouchsafed to us in the light of close and accurate observation of the phenomena of everyday life, allowing chemistry to confirm what it may, but to originate nothing.

What the Reviewer says of the physiological effects of alcohol, as to its hydro-carbon supplying fuel to the system, is true only in the abstract. That it does supply fuel more promptly than food, and more largely too, bulk for bulk, is not denied, but that such prompt and large supply is healthful, is another question; and is the very thing to be proven. On this point the whole controversy turns. The system cannot subsist without hydro-carbon; alcohol is hydro-carbon in its concentrated form; no logician would say, "*therefore alcohol is essential to the system*," much less "*healthful* to the system." It would be as perfect a non sequitur, as that pure oxygen is necessary to the system and healthful to it, because it is assuredly true that "without oxygen the system could not subsist at all." The truth is, every physiologist and every chemist knows, that the human body will die in an hour without oxygen, but he also knows that if pure oxygen is administered—concentrated oxygen, unadulterated oxygen—death is no less certain within a very few hours. The same line of argument obtains as to alcohol, or hydro-carbon. Without it, the human body would not live a day; and in proportion as it is used in its purity, in its concentrated form, just in such proportion and certainly will the man die.

It is not in their purity, in their concentrated form, that the life and health-giving elements of our physiological existence are ever supplied us, but in their very large dilution with other ingredients, just as necessary. Sugar is carbon in a very large proportion, and without carbon we cannot live a day or an hour; and yet the man who would eat only sugar, would certainly die in a few days. This single argument is subversive of all that is said in that elaborate and mischievous article. Mischievous, because there is just enough of science in it to convince the superficial, and to fortify the unreflecting in following practices which they love. While it is a perfect God-send to those who have a pride in having it believed that they are among the few, who are wise enough to be *conservative*, to choose the mean.

Sober reason would tell us that alcohol, largely used, would destroy health and life, if we had never seen a single instance of health and life destroyed by it, because in our observation, we know of no element necessary to the health of the human body, which would not subvert that health, if used largely, or exclusively in its concentrated form. Sugar, its hyro-carbon, is essential to life, and more or less of it is found in every article of food; the same with starch; but either of these articles alone, or in aliments mainly made up of them, will not sustain health long, and why should we suppose alcohol would be an exception to an otherwise universal law?

Nature has formed no element in its purity, which is necessary to physiological life, and we have no reason to suppose that such pure element would contribute to human health, when artificially fabricated. As millions have lived to a great age without the use of alcohol, that is a demonstration of its not being essential to health. And as men do live in health without alcohol, we may conclude that alcohol does not promote health in those who are well, because a man cannot be more than well.

The general summing up is this.

If alcohol is not a poison, but food; because alcohol gives force, muscular power—then, arsenic is not a poison, but food, because arsenic gives force, muscular power.

As nature has formed no element in its purity, which element in large dilution is necessary to health, we conclude that such element in its purity is not essential to health; therefore alcohol is not essential to health.

As men have lived in perfect health without alcohol, the use of alcohol cannot add to that health, because a man cannot be better than well.

As we know of no article which contains hydro carbon largely, which would not destroy life, if used alone, not even sugar; so we may conclude that alcohol, which does contain hydro-carbon largely, will destroy life, if used alone.

If any elementary substance in its purity destroys life, if used alone, it is reasonable to conclude that the only safe method of using any elementary substance, is, in using it in the proportion in which nature has combined it with other materials: therefore, that however essential to existence hydro-carbon may be, it is not healthful or safe to use it in its concentrated, artificial combination, but only healthful and safe in deriving our supplies of it, as contained in our natural food. Therefore, we consider it a *Q. E. D.*, that alcohol is not essential to health; that it is not promotive of the health of those who are well; and that in proportion as it is used largely, or alone, in such proportion is it, like all other elementary concentrations, certainly destructive of health and life together.

